# A Plea for a Greater Use of Non-Cohesive Gold

**Published:** 1897-08

**Authors:** J. N. Crouse

**Affiliations:** Chicago


### SELECTIONS.

### A Plea for a Greater Use of Non-Cohesive Gold.

BY J. N. CROUSE, D.D.S , CHICAGO.

Having read various papers on the use of gold and the filling of proximal cavities, I have been alarmed at the apparent disuse of an old but very reliable method, namely, the use of non-cohesive gold in the form of tightly-rolled cylinders, which is the wedge principle. These cylinders are best made from No. 3 or 4 gold foil, which should be non-cohesive. If it has any cohesiveness, that should be removed by placing the foil in a drawer with ammonia for a few hours. To make the cylinders, fold the gold foil upon itself until you have a ribbon a little wider than the depth of the cavity at the cervical margin. Having filed a broach to a triangular peak, lay it on one end of the ribbon and turn gently when the gold will be wrapped around the broach, making cylinders a little more in width than the depth of the cavity.

These cylinders are made differently for various cases. In large cavities the first one or two may contain a sheet of No. 3 foil, the rest less; some rolled quite tightly on the broach, others less so. With a variety of cylinders thus prepared we are ready to fill well a large proportion of the cavities which occur on the proximal and buccal surfaces of bicuspids and molars, and in one-fourth the time required to make as safe a filling with cohesive gold.

. About the same amount of time and painstaking effort is needed in either case to prepare the cavity, but it is shaped somewhat differently for non-cohesive gold, and especially for cylinders as described. There are no pits drilled in the dentine from which to start the filling, and the cervical walls need be but little undercut, as strength of anchorage when the cavity is completed is depended upon toward the grinding surface, this being the point where the greatest strain comes on the filling by force of mastication. The lateral walls usually have sufficient undercut when the decay is removed, or if not, slight grooves or undercuts should be made.

With the cavity prepared, and where the walls are of good strength, it is a waste of time to use cohesive gold for any except the latter part of the filling, and then only in cases where it includes part of the grinding surface; select a cylinder which when placed lengthwise will extend a little beyond the cervical margin, or in cases of large and deep cavities one large and long enough to rest against the opposing tooth and to entirely cover the cervical margin, and start the filling. In extreme cases two sheets of No.2 made into one cylinder can be used to good advantage. Generally, however, a large cylinder on each side and a smaller one between the two make a good foundation. Always remember at this point not to condense each cylinder separately, thus crowding them apart, but a place should be made for the next one, which should be rolled tighter and of a size that will occupy the place as a wedge. Continue until the cavity is about two-thirds full, when, if it is required, cohesive gold can be driven into and between the cylinders at different points, and then the whole mass of gold, being for the most part non-cohesive and so quite ductile, may be forced into every crevice, making a very perfect filling. On top of the cohesive gold already in the cavity we can readily add more and so continue the filling over and on to the grinding surface, contour and finish.—*Dental Digest.*

